# Accurate microRNA target prediction correlates with protein repression levels

**DOI:** 10.1186/1471-2105-10-295

**Published:** 2009-09-18

**Authors:** Manolis Maragkakis, Panagiotis Alexiou, Giorgio L Papadopoulos, Martin Reczko, Theodore Dalamagas, George Giannopoulos, George Goumas, Evangelos Koukis, Kornilios Kourtis, Victor A Simossis, Praveen Sethupathy, Thanasis Vergoulis, Nectarios Koziris, Timos Sellis, Panagiotis Tsanakas, Artemis G Hatzigeorgiou

**Affiliations:** 1 Institute of Molecular Oncology, Biomedical Sciences Research Center 'Alexander Fleming', Vari, Greece; 2 Institute of Computer Science, Martin Luther University Halle-Wittenberg, 06120 Halle, Germany; 3 School of Biology, Aristotle University of Thessaloniki, Thessaloniki 54124, Greece; 4 Synaptic Ltd., Heraklion, Greece; 5 Institute for the Management of Information Systems, "Athena" Research Center, Athens, Greece; 6 Knowledge and Database Systems Lab, Department of Computer Science, School of Electrical and Computer Engineering, National Technical University of Athens, Greece; 7 Computing Systems Laboratory, Department of Computer Science, School of Electrical and Computer Engineering, National Technical University of Athens, Greece; 8 Genome Technology Branch, National Human Genome Research Institute, National Institutes of Health, Bethesda, MD, 20876, USA; 9 Department of Computer and Information Sciences, University of Pennsylvania, Philadelphia, PA, USA

## Abstract

**Background:**

MicroRNAs are small endogenously expressed non-coding RNA molecules that regulate target gene expression through translation repression or messenger RNA degradation. MicroRNA regulation is performed through pairing of the microRNA to sites in the messenger RNA of protein coding genes. Since experimental identification of miRNA target genes poses difficulties, computational microRNA target prediction is one of the key means in deciphering the role of microRNAs in development and disease.

**Results:**

DIANA-microT 3.0 is an algorithm for microRNA target prediction which is based on several parameters calculated individually for each microRNA and combines conserved and non-conserved microRNA recognition elements into a final prediction score, which correlates with protein production fold change. Specifically, for each predicted interaction the program reports a signal to noise ratio and a precision score which can be used as an indication of the false positive rate of the prediction.

**Conclusion:**

Recently, several computational target prediction programs were benchmarked based on a set of microRNA target genes identified by the pSILAC method. In this assessment DIANA-microT 3.0 was found to achieve the highest precision among the most widely used microRNA target prediction programs reaching approximately 66%. The DIANA-microT 3.0 prediction results are available online in a user friendly web server at

## Background

MicroRNAs (miRNAs) are short, endogenously expressed RNA molecules that regulate gene expression by binding directly and preferably to the 3' untranslated region (3'UTR) of protein coding genes [1]. Each miRNA is 19-24 nucleotides in length and is processed from a longer transcript which is referred to as the primary transcript (pri-miRNA). These transcripts are processed in the cell nucleus to short, 70-nucleotide stem-loop structures known as pre-miRNAs. Pre-miRNAs are processed to mature miRNAs in the cytoplasm by interaction with the endonuclease Dicer which cleaves the pre-miRNA stem-loop into two complementary short RNA molecules. One of these molecules is integrated into the RISC (RNA induced silencing complex) complex and guides the whole complex to the mRNA, thus inhibiting translation or inducing mRNA degradation [2]. Since their initial identification, miRNAs have been found to confer a novel layer of genetic regulation in a wide range of biological processes. miRNAs were first identified in 1993 [3] via classical genetic techniques in C. elegans, but it was not until 2001 that they were found to be widespread and abundant in cells [4-6]. This finding served as the primary impetus for the development of the first computational miRNA target prediction programs. DIANA-microT [7] and TargetScan [8] were the first algorithms to predict miRNA targets in humans, and led to the identification of an initial set of experimentally supported mammalian targets. Such targets are now collected and reported in TarBase [9] which contains more than one thousand entries for human and mouse miRNAs.

**Figure 1:**
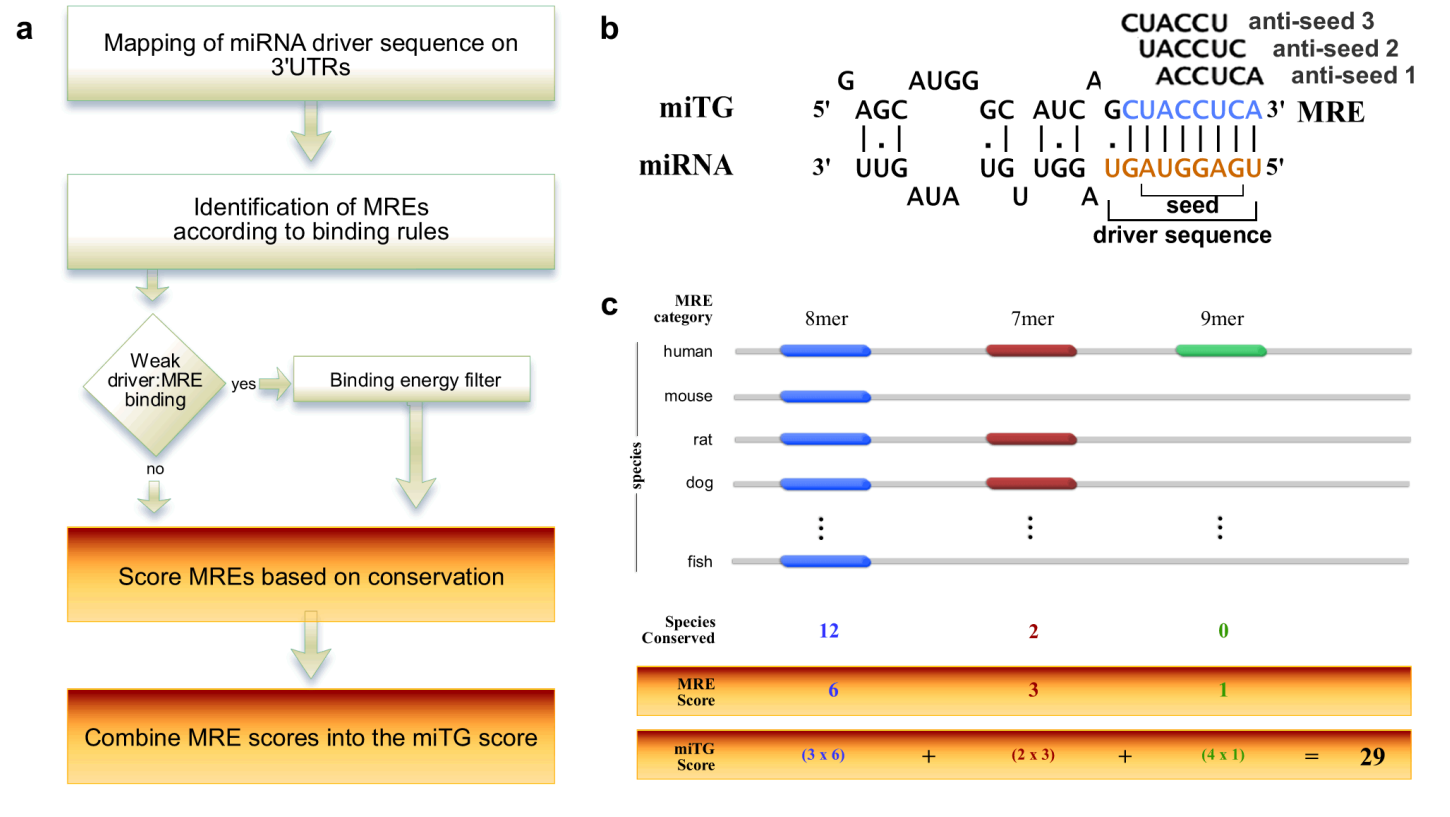
**The DIANA-microT 3.0 algorithm**. (a) A schematic overview of the algorithm. The miRNA driver sequence is mapped onto a 9 nt length window that slides along the 3'UTR sequence. The binding category of the driver:MRE interaction is defined by the number of binding nucleotides between the two sequences. G:U wobble pairs or less than 7 consecutive WC matches are only allowed if the free binding energy of the miRNA:MRE heteroduplex is under a binding category specific threshold (lower free binding energy corresponds to stronger binding). MREs are scored according to their binding category and degree of conservation in other species. The final miTG score is the weighted sum of all MREs on the miTG. (b) The top sequence (MRE) is part of the 3'UTR of a gene. The nine nucleotide region near the 5'end of the miRNA is called the driver sequence of the miRNA (shown in red). Sequences on the MRE, corresponding to positions 1-6, 2-7 and 3-8 from the miRNA 5'end are called anti-seed 1, anti-seed 2 and anti-seed 3 respectively. (c) An example of the miTG score calculation. The top line represents the 3'UTR sequence of a human gene containing three MREs with different conservation levels. Individual MRE scores are calculated depending on the degree of conservation of the MRE, and multiplied by a weight depending on the MRE binding category. The sum of all weighted MRE scores defines the final miTG score.

In the last years several groups suggested that the first nucleotides of a miRNA sequence are crucial for recognizing and binding to the messenger of a protein. Kiriakidou *et al. *[7] showed the need for a nearly consecutive binding of the first 9 miRNA nucleotides (*driver *sequence) (figure 1b) to the 3'UTR of protein coding genes in order to repress translation. A statistical approach by Lewis et al. [10] revealed that complementary motifs to nucleotides 2-7 of the miRNA driver sequence (miRNA *seed *region) remain preferentially conserved in several species. Typically, it is believed that a binding of at least 7 consecutive Watson-Crick (WC) base pairing nucleotides between the miRNA driver sequence and the miRNA Recognition Element (MRE) is required for sufficient repression of protein production. However, experimental evidence [11] show that weaker bindings, involving only six consecutively paired nucleotides or including imperfect bindings (e.g. G:U wobble, bulge) may also confer protein repression although they might generally be less effective [12]. For this reason, miRNA target prediction programs mostly rely on sequence alignment of the miRNA seed region to the 3'UTR sequences of candidate target genes in order to identify putative miRNA binding sites. Their specificity is usually increased by additionally assessing the commonly observed binding site evolutionary conservation or by using additional features such as binding site structural accessibility [13,14], nucleotide composition flanking the binding sites [15] or proximity of one binding site to another within the same 3' UTR [12,15,16].

DIANA-microT 3.0, the algorithm described here, utilizes the above mentioned features and categorizes as putative MREs those sites that have seven, eight or nine nucleotide long consecutive WC base pairing with the miRNA driver sequence, starting from position 1 or 2 of the 5'end of the miRNA. For sites with additional base pairing involving the 3'end of the miRNA, a single G:U wobble pair or binding of only 6 consecutive nucleotides to the driver sequence are allowed. Briefly, the DIANA-microT 3.0 algorithm consists of (figure 1a): a) alignment of the miRNA driver sequence on the 3'UTR of a protein coding gene, b) identification of putative MREs based on specific binding rules, c) scoring of individual MREs according to their binding type and conservation profile, d) calculation of an overall miRNA target gene (miTG) score through the weighted sum of all MRE scores lying on the 3'UTR. The program is designed to use up to 27 different species to estimate MRE conservation scores and combines both conserved and non-conserved MREs in a final miTG score (figure 1c). The miTG score correlates with fold changes in protein expression. Additionally, since the algorithm calculates all weights and scores independently for each miRNA it allows for the calculation of signal to noise ratio (SNR) at different miTG score cut-offs providing precision scores which serve as an indication of the false positive rate of the predicted interactions.

Generally, miRNAs can repress the expression of proteins in two ways: via mRNA degradation or via repression of mRNA translation. Until recently, high throughput experiments were only able to measure miRNA-mediated changes at the mRNA level (degradation), allowing the characterization of only a subset of direct miRNA targets [17,18]. However, recently two groups [12,19] have independently developed methods to characterize miRNA-mediated gene expression changes at both the mRNA and the protein level. Selbach *et al. *[19] used microarrays and pulsed stable isotope labeling with amino acids in cell culture (pSILAC) assays to determine the genes targeted by each of five over-expressed miRNAs in HeLa cells. Using this set of experimentally supported targets the authors performed a comparative assessment of several target prediction programs. The benchmark revealed that the simplest prediction method involving the search for complementary sequences of the miRNA seed region on the 3'UTR of genes achieved a precision (the fraction of the predicted targets that were actually downregulated) of 44% while only three of the prediction programs (including an initial version of DIANA-microT 3.0) achieved significantly higher precision. PicTar [20] and TargetScanS [10] achieved approximately 62% precision compared to DIANA-microT 3.0 with approximately 66%.

## Methods

### Identification of putative miRNA binding sites through sequence alignment

The program identifies the highest scoring alignment between every nine nucleotide long window of the 3'UTR with the miRNA driver sequence using a dynamic programming algorithm. The alignment is based on the following binding rules. Firstly, a minimum of six consecutive matches (Watson-Crick (W-C) or G:U) is required. If the six matches are W-C and the binding starts at position 1 or 2 of the miRNA driver sequence, then the MRE is considered a 6mer. A 7mer (8mer, 9mer) has seven (eight, nine) consecutive W-C matches starting at position 1 or 2 of the miRNA driver. A single G:U wobble pair is allowed as long as there are at least six W-C pairs, yielding 7mers, 8mers and 9mers, each with a wobble base pair.

### Filter of putative miRNA binding sites depending on binding energy

**Figure 2:**
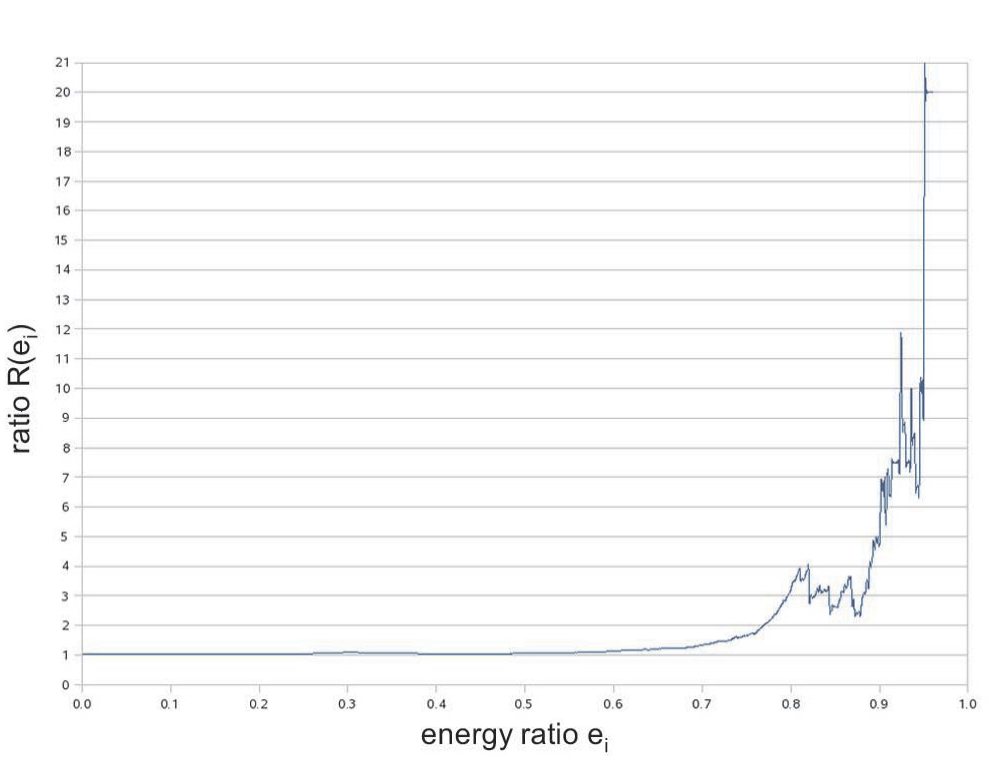
**Hybridization energy ratio**. Ratio *R*(*e*_*i*_) (vertical axis) is plotted against the energy ratio *e*_*i *_(horizontal axis). The curve corresponds to the binding category which consists of seven WC pairs and a single G:U wobble pair.

For sites with less than 7 consecutive W-C matches (6mer, 7mer with wobble, 8mer with wobble, 9mer with wobble) an additional energy filter is applied. Using RNAhybrid [21] the algorithm estimates the free binding energy between the miRNA sequence and the 3'UTR sequence flanking the identified putative binding site and compares it to the perfect complement energy of the miRNA. As "perfect complement energy" we denote the hypothetical energy of the perfect binding between the miRNA sequence and its reverse complement sequence. Therefore an imperfect site, in terms of alignment, is considered as MRE only if the ratio of the free binding energy to the perfect complement energy is higher than a binding-category specific threshold. A threshold of 0.6 is used for 9mers and 8mers containing a G:U wobble pair, and a threshold of 0.74 is used for 7mers with a G:U wobble pair and 6mers. The energy thresholds have been calculated by comparing the predicted binding sites of the real miRNA sequence versus the predicted binding sites of several shuffled miRNA sequences. The shuffled miRNA sequences are designed to have the same driver as the real miRNA but a shuffled 3' end with the same nucleotide composition as the real miRNA. The free binding energy ratio *e*_*i *_is defined as the ratio of the free binding energy between the miRNA sequence and the 3'UTR sequence flanking the identified putative binding site over the miRNA perfect complement energy. Additionally, *N*_*r*_(*e*_*i*_) is defined as the number of binding sites of the real miRNAs that have energy ratios greater than *e*_*i *_and as *N*_*S*_(*e*_*i*_) the number of binding sites of the shuffled miRNAs that have energy ratios greater than *e*_*i*_. The ratio *R*(*e*_*i*_) = *N*_*r*_(*e*_*i*_)/*N*_*S*_(*e*_*i*_) indicates how much more prevalent the free binding energy *e*_*i*_for real binding sites compared to the shuffled ones is. An example of the way this ratio *R*(*e*_*i*_) fluctuates is provided in figure 2. For each binding category the energy thresholds have been chosen at the point where the ratio *R*(*e*_*i*_) becomes greater than 2 indicating that at this energy value one can generally find two times more real binding sites than random binding sites.

### Mock miRNAs

Mock miRNAs are artificially produced miRNA sequences which are independently created for each real miRNA. These artificial miRNA sequences are designed to have approximately the same number of predicted MREs as the corresponding real miRNA and are generated through the following procedure. Initially, all 3'UTR sequences are scanned for sites perfectly complementary to each possible 6 nucleotide long motif (hexamer) excluding those motifs corresponding to positions 1-6, 2-7 and 3-8 of real miRNAs. The 60 hexamers having the closest number of complementary sites to those of the seed of the real miRNA are chosen. These hexamers are then used as the seed of each artificially created mock miRNA. The remaining sequence of the mock miRNAs is then produced by randomly shuffling the remaining nucleotides of the real miRNA.

### miRNA Recognition Elements score (MRE score)

**Figure 3:**
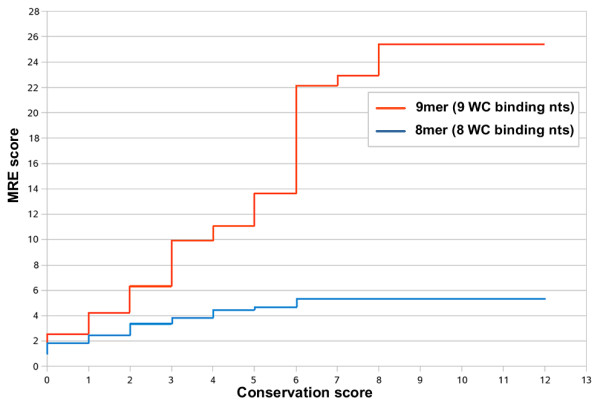
**miRNA recognition element score**. The MRE score (vertical axis) is plotted against the MRE conservation score (horizontal axis) for two different binding categories.

The identified MREs are checked for sequence conservation in several species based on the sequence alignment of ortholog UTRs. An MRE X is considered conserved in species A if X can also be identified at the exact same position on the ortholog 3'UTR sequence of species A. The conservation score *c*of an MRE is defined as the number of species in which the MRE is conserved. The MRE score is calculated individually for each real miRNA *r*, each binding category *b *and each conservation score *c*. Analytically, for each binding category the number of MREs *N*_*r*, *b*_(*c*) of the real miRNA and the number of MREs *M*_*r*, *m*, *b*_(*c*) of the corresponding mock miRNAs with conservation score equal or greater than *c *are counted and the ratio of the two defines the MRE score (of binding category *b *at conservation score *c*). The equation defining this procedure is  in which *r *is the index of the real miRNA, *b *corresponds to the binding category, *c *defines the conservation score and *m *defines the index of the mock miRNA from the set of mock miRNAs corresponding to the real miRNA *r*. In the described procedure the ratio is kept constant if *N*_*rb*_(*c*) or *M*_*r*, *m*, *b*_(*c*)/60 become less than 20. Figure 3 shows an example of *R*_*rb *_for 2 binding categories at different MRE conservation scores.

### miRNA target gene score

**Figure 4:**
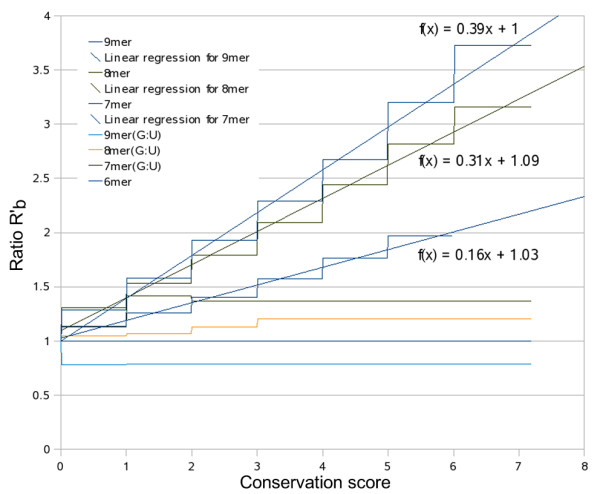
**Binding categories differ from the mock background**. Ratio  (vertical axis) versus the conservation score (horizontal axis) for the set consisting of 75 miRNAs conserved in human, chimp, mouse, rat, dog, chicken. This diagram indicates how each binding category may be differentiated as the conservation score increases (more conserved MREs). It may be seen that 9mers tend to differentiate more than 8mers and 8mers more than 7mers. Except for categories "9mer", "8mer" and "7mer" the remaining categories do not seem to differ significantly from the background.

The scores of the MREs identified on the same 3'UTR are combined through a weighted sum to produce the final miTG score. The weights *w*_*b *_for each binding category *b *are calculated using 75 miRNAs conserved in human, chimpanzee, mouse, rat, dog and chicken, by comparing them to 375 mock sequences (5 mock miRNAs for each miRNA). The analysis is similar to the calculation of the MRE score explained previously but in this case the 75 miRNAs are not treated independently but as a total. The ratio  for binding category *b *and conservation score *c *is calculated as  where *N*_*rb*_(*c*) is the number of MREs of the *r *real miRNA categorized to binding category *b *and having a conservation score greater than *c*, *M*_*r*, *m*, *b*_(*c*) represents the number of MREs of the *m *mock miRNA categorized to binding category *b *succeeding a conservation score greater than *c *and corresponding to real miRNA *r*. As shown in figure 4 the weights for each binding category are estimated based on the slope of a fitted line. Fitting is performed based on linear least squares approximation. For each binding category the weight is defined as *w*(*bindingcategory*) = *slope*(*bindingcategory*)/*slope*(9mer). For example, the weight for category "8mer" would be w_8mer _= 0.31/0.39 = 0.79. Except for "9mer", "8mer" and "7mer" the remaining categories do not differ significantly from the mock background and consequently in this analysis no specific weights are calculated for these categories. In order to approximate the estimated weights *Dw*_*b *_based on the above analysis, each MRE score is multiplied by a specific weight *mw*_*b *_which depends on the binding category of the MRE (table 1).

**Table 1:** Binding category weights

**Category**	**Estimated Weights (w_*b*_)**	**Multiplication weights (mw_*b*_)**	**Overall Diana weights *Dw*_*b *_= *mw*_*b*_/*mw*_9*mer*_**
9mer	1	4	1.00 = 4/4

8mer	0.79	3	0.75 = 3/4

7mer	0.41	2	0.50 = 2/4

other	-	1	0.25 = 1/4

The binding weights estimated for each binding category and the weights used in DIANA-microT 3.0.

### miTG score threshold assessment

**Figure 5:**
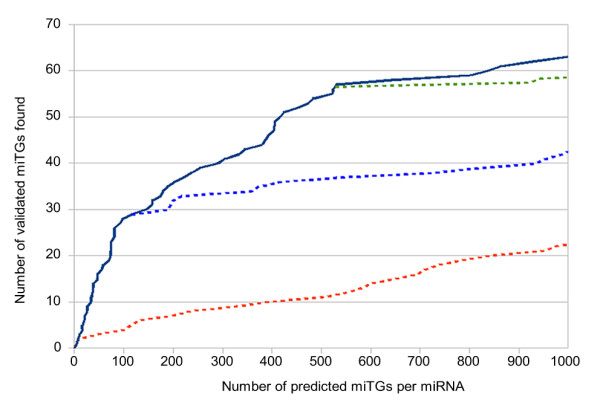
**Define biologically meaningful score threshold**. Experimentally validated targets correctly predicted by DIANA-microT 3.0 versus the average number of predicted miTGs per miRNA. The slope of this curve corresponds to the rate in which correct validated targets are discovered as more miTGs are predicted. There are two distinct points in which the slope changes. These points correspond to miTG score values of 19 and 7.3 which are proposed as the strict and loose miTG score thresholds respectively. As a control, the order of miTGs with scores lower than each threshold was shuffled. The discovery rate of these controls is shown with dotted lines. The red line shows all miTGs in random order, the blue line those with miTG score under 19 and the green line those with miTG score under 7.3. The difference in slope between the solid line and each dotted line shows the improvement on the discovery rate achieved by the DIANA-microT scoring scheme. Two other target prediction programs (Pictar and TargetScan 4.2) have been compared to DIANA-microT 3.0 on the same dataset achieving similar precision levels (figure 9).

A common challenge among miRNA target prediction programs is the decision on a score threshold that will reduce the number of misclassifications. Here a set of 100 experimentally supported targets for 43 different human miRNAs, provided by TarBase 5.0 [9], has been used in order to determine a biologically meaningful score threshold. Based on this dataset, an analysis was performed to test the capability of the algorithm to identify supported targets when increasing the miTG score threshold. As expected, the algorithm's capability reduces as the miTG score increases (figure 5). However, there are two distinct miTG scores (7.3 and 19.0) with significantly higher performance reduction. For this reason, these miTG score values have been chosen as a loose and strict miTG score threshold respectively. However, users are still allowed to adjust the threshold at will to exchange between specificity and sensitivity levels.

### Precision

The precision of a prediction is defined as the ratio of correct positive predictions over all positive predictions [*precision *= *truepositive */(*truepositive *+* falsepositive*)]. In the case of DIANA-microT 3.0, the average number of miTGs for mock miRNAs provides an estimation of the number of false positive targets predicted. Therefore, the number of true positive predicted miTGs can be calculated by subtracting the average number of predicted miTGs for the mock miRNAs from the total number of predicted miTGs for the real miRNA. In detail, the precision for miRNA *r *at miTG score *s *is calculated by  where W_*r *_is the number of miTGs of the *r *real miRNA having miTG scores from *s *to *s *+ Δ*s*,  is the average number of miTGs of the mock miRNAs corresponding to miRNA *r *having miTG scores from *s *to *s *+ Δ*s *and Δ*s *is a specified miTG score window (Δ*s *= 3).

### miRNA sequences

The human and mouse miRNA sequences used by DIANA-microT 3.0 have been downloaded from miRBase Build 10.0 [22].

### 3'UTR sequences

The gene 3'UTR sequences have been downloaded from Ensembl, release 48 [23]. Those 3'UTR sequences that correspond to the same gene but to different gene transcripts have been filtered to keep only the longest 3'UTR sequence.

### Multiple Alignment Files (MAFs)

The multiple genome alignment files have been downloaded from the UCSC Genome Browser [24]. The file used for human (hg18) is the alignment to 16 vertebrate genomes while for mouse (mm9) 29 vertebrate genomes are used.

## Results

### Signal to Noise Ratio (SNR) assessment

**Figure 6:**
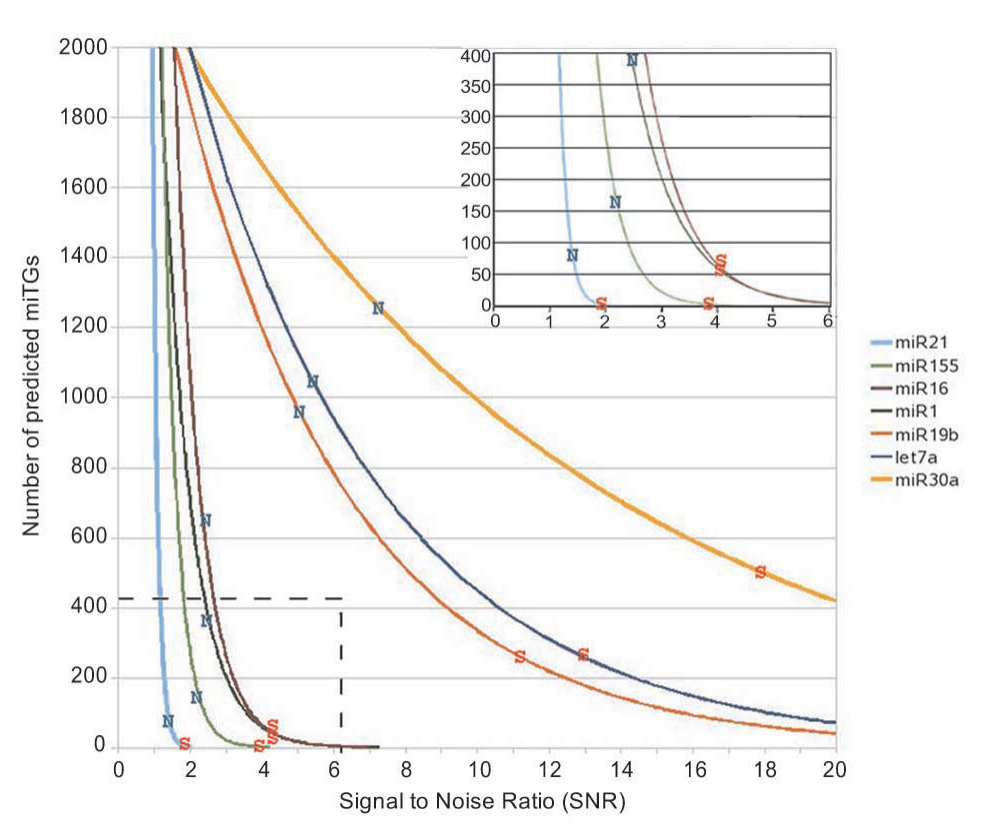
**Signal to noise ratio for 7 miRNAs**. Curves showing the number of predicted miTGs versus the SNR for 7 miRNAs. The loose and strict thresholds have been marked in the figure with the symbols "N" and "S" respectively.

**Figure 7:**
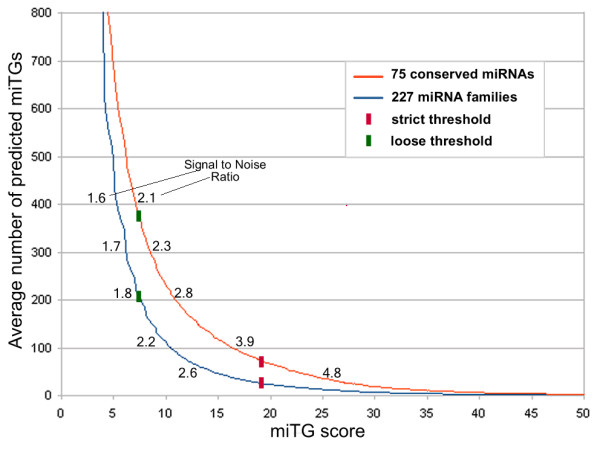
**Overall signal to noise ratio**. The mean number of predicted miTGs per miRNA for different miTG score cutoffs. The red curve corresponds to a set of 75 miRNAs conserved in at least six species (human, chimp, mouse, rat, dog, chicken), whereas the blue curve corresponds to a set of 227 miRNAs which represent the miRNA families (with varying conservation levels). The values next to the curves indicate the overall SNR. Higher miTG score leads to fewer predicted miTGs with higher overall SNR, which suggests a lower number of false positive predicted miTGs. The suggested strict (red bars) and loose (green bar) miTG score thresholds are marked on the curves. For the strict miTG score threshold (miTG score = 19), the estimated overall SNR for the set of 227 miRNAs (blue line) is 3, meaning that approximately one in three predicted miTGs might be a false positive. In comparison, at the loose suggested threshold (miTG score = 7.3), approximately one in two predicted miTGs might be a false positive.

The signal to noise ratio for a prediction algorithm is typically used for the evaluation of its specificity. For DIANA-microT 3.0 the overall SNR is defined as the average signal to noise ratio calculated individually for each miRNA. The individual miRNA signal to noise ratio calculation is performed by dividing the number of predicted miTGs of a real miRNA by the number of predicted miTGs for the set of corresponding mock miRNAs. It is assumed that the predicted miTGs for the mock miRNA sequences provide an unbiased estimate of the number of miTGs predicted by chance alone. Analytically, the SNR value of miRNA *r *at miTG score *s *is calculated as . In this formula *NG*_*r*_(*s*) refers to the number of miTGs of the real miRNA *r *having miTG scores greater than *s *while *MG*_*r*, *m*_(*s*) refers to the number of miTGs of the mock miRNA *m *corresponding to the real miRNA *r *having miTG score greater than *s*. Figure 6 presents a graph of the *SNR *for seven different miRNAs. The overall SNR calculation for DIANA-microT 3.0 is performed on two different sets of miRNAs. The first set consists of 75 miRNAs conserved in 6 vertebrate species while the second set consists of 227 unique miRNAs each one representing a miRNA family with varying conservation levels. Figure 7 shows the diagram for the number of predicted miTGs versus the miTG score. For an miTG score threshold that yields an average of approximately 100 predicted target genes per miRNA, DIANA-microT 3.0 achieves an overall SNR of 3.9 for the first dataset and an overall SNR of 2.2 for the second dataset which indicates that conserved miRNAs tend to achieve higher SNR values.

### Receiver Operating Characteristics (ROC) analysis on proteomics data

**Figure 8:**
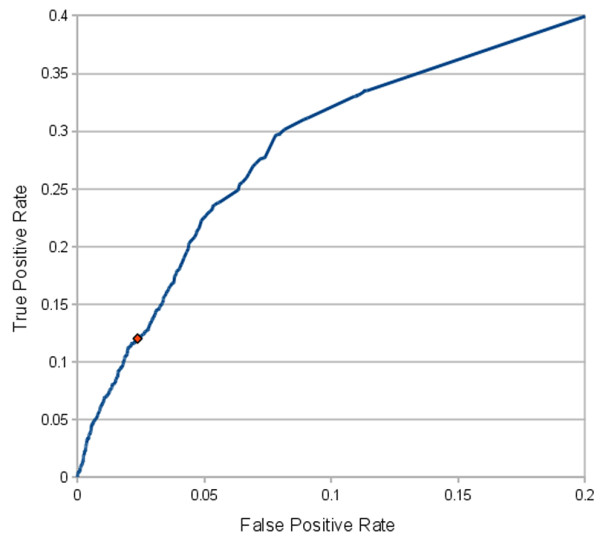
**DIANA-microT 3.0 ROC curve**. The ROC curve for DIANA-microT 3.0 calculated on the pSILAC data [19]. The suggested loose threshold of DIANA-microT 3.0 has been marked on the diagram with a red dot.

Until recently a common difficulty in assessing the performance of a prediction algorithm was that the available experimental data could not easily distinguish between true and false targets. However, the recent study of Selbach *et al. *provides both classes of targets allowing for the estimation of both the true positive rate as well as the false positive rate of a prediction. Using a log_2 _fold change cutoff of -0.2 to distinguish between targeted and non-targeted genes, the performance of DIANA-microT 3.0 is assessed and presented as a ROC curve (figure 8).

### Correlation of miTG score to the repression of protein production

**Figure 9:**
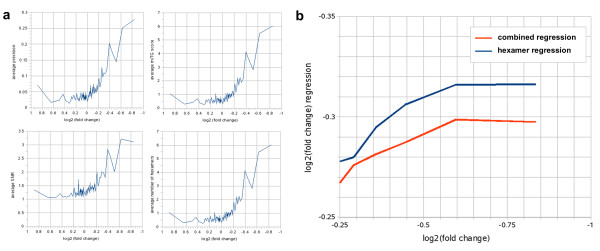
**Correlation of DIANA-microT 3.0 prediction measures to protein repression**. Fold changes are calculated for approximately 5,000 proteins after overexpression of a miRNA. The results for five miRNAs, as provided by Selbach et al., are used. The fold change and the miTG score is averaged in groups of 150 proteins sorted by fold change. (a) The correlation of several miRNA target prediction measures with protein production fold change induced by the same miRNAs. It may be observed that there is a trend for values of all the measures to increase as the level of downregulation increases. (b) The red line indicates the correlation between the anti-seed 2 occurrences on the 3'UTRs of downregulated genes with the protein production fold change of the corresponding genes using a linear regression. The blue line shows the corresponding correlation for a linear regressor based on a combination of the miTG score, the precision, the SNR and the anti-seed 2 frequency. The combined linear regressor correlates better with the protein production fold change than the regressor based solely on the anti-seed 2 frequency.

In the study by Selbach *et al*[19], it was observed that there is a correlation between the log_2_-fold change of protein production with the number of occurrences of the hexamer corresponding to the seed of a miRNA in the 3'UTR of downregulated genes. When investigating the same data using DIANA-microT 3.0, a similar correlation between the level of protein down-regulation and the predicted miTG scores, SNR, and precision values is observed (figure 9a). Interestingly, a linear regression analysis shows that the combination of miTG score, precision, SNR, and the number of anti-seeds (regions on the gene 3'UTR complementary to the motifs 1-6, 2-7, 3-8 of the miRNA) as regressors provides the best accuracy in attempting to predict such fold changes in protein expression. Figure 9b demonstrates the relationship between the protein expression fold change versus the number of occurrences of the miRNA anti-seed 2 (adjusted R^2 ^= 0.12) as well as the protein expression fold change versus the combined regressor (adjusted R^2 ^= 0.15).

## Discussion and conclusion

**Figure 10:**
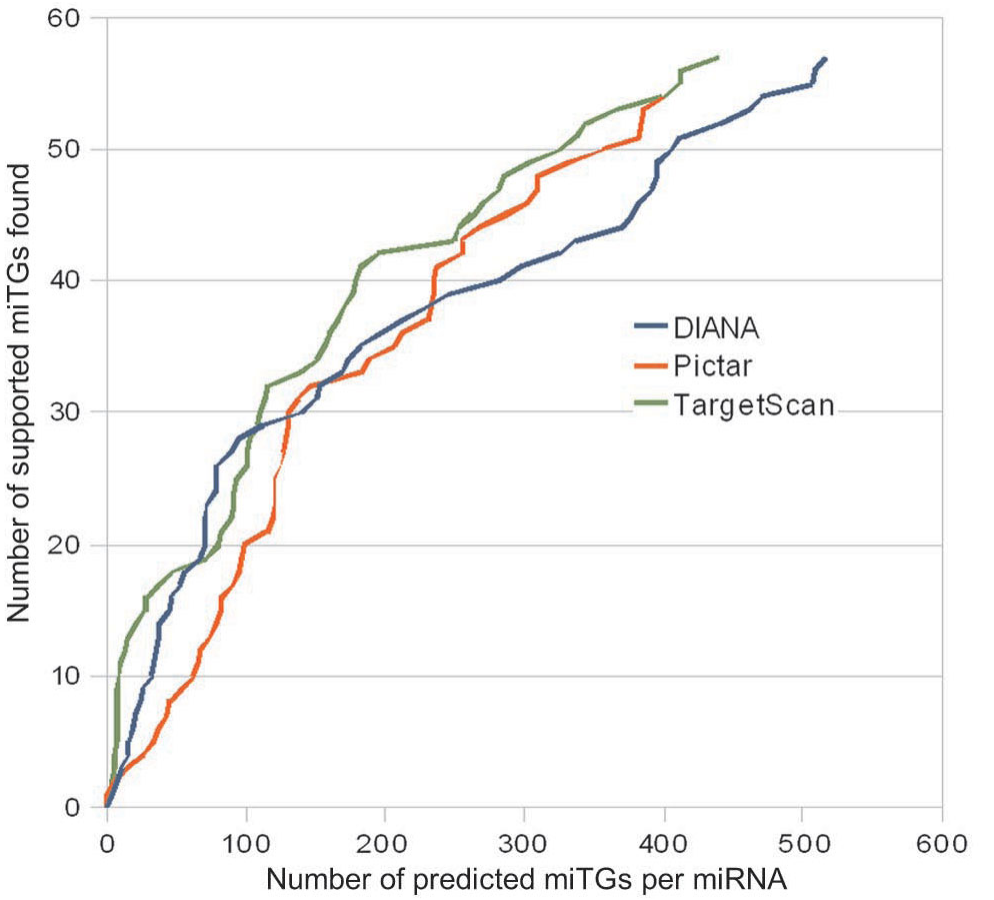
**Comparison on experimentally supported targets**. Comparison of three target prediction programs (DIANA-microT 3.0, Pictar and TargetScan 4.2) on the experimentally supported dataset. The average number of predicted miTGs per miRNA is presented on the horizontal axis. The total number of correctly predicted experimentally validated targets is shown on the vertical axis. All three programs tested perform similarly.

In the last five years more than two dozen miRNA target prediction programs for mammalian genomes have been published [25]. Using data from a high throughput experiment on five miRNAs [19] as a true-positive set of targets, it has been shown that DIANA-microT 3.0 achieves comparable precision to two other leading target prediction programs, TargetScanS [8] and PicTar [20]. Additionally, DIANA-microT 3.0 provides prediction scores which correlate with protein production fold change and may be used as an indication of the expected fold change in protein production. The performance of the algorithm has been analyzed further by using a different set of supported miRNA targets which has been extracted by the database of experimentally supported targets [9]. The results also indicate that the three programs (DIANA-microT 3.0, PicTar and TargetScan 4.2) achieve similar precision levels (figure 10). However, as shown in table 2 and 3 there are significant differences among the miTGs predicted by DIANA-microT 3.0 and those predicted by each of the other programs. Table 3 indicates that only 40% of the miTGs predicted by DIANA-microT 3.0 are also predicted by PicTar, and only 48% are predicted by TargetScan 4.2. This leaves in either case approximately 50% of the targets predicted only by DIANA-microT 3.0.

**Table 2:** Number of miTGs predicted in common by programs

	**Diana-microT**	**PicTar**	**TargetScan 4.2**
Diana-microT	**22391**	8882	10651

PicTar		**17135**	12902

TargetScan 4.2			**19299**

The table diagonal corresponds to the total number of miTGs predicted by each program for all the miRNAs which are included in the set of experimentally verified targets. The number of miTGs predicted in common by each two target prediction programs is shown in the table. For example, TargetScan and PicTar have 12902 predicted targets in common while DIANA-microT and PicTar have 8882.

**Table 3:** Percentage of common predictions among programs

	**Diana-microT**	**PicTar**	**TargetScan 4.2**
Diana-microT	**100%**	39.67%	47.57%

PicTar	51.84%	**100%**	75.30%

TargetScan 4.2	55.19%	66.85%	**100%**

The percentage of each program's predicted targets (rows) which are also predicted by another program (columns) for all the miRNAs which are included in the set of experimentally verified targets. For example, from the miTGs predicted by DIANA-microT 3.0, 39.67% are also predicted by PicTar and 47.57% by TargetScan 4.2.

Recently, the rapid growth in the discovery rate of novel miRNA sequences due to extensive usage of deep sequencing technology [14], and the fact that miRNAs have been shown to undergo A-to-I RNA editing [15] have underlined the need for a web based program which would allow for miRNA target predictions based on user defined miRNA sequences. DIANA-microT 3.0 is one of the few programs offering such a service, supporting the scientific community with a tool which in total can be extensively used for the analysis of miRNA dependent processes. This tool can be accessed thought the DIANA-microT [26] web server at  which includes an optimized prediction algorithm that provides several features, combined with a user friendly interface which assists in the identification of interactions of interest.

As already mentioned, DIANA-microT 3.0 takes into account both conserved and not conserved MREs. This attribute provides the algorithm with a highly important capability to predict targets of viral miRNA sequences. Generally, targets of viral miRNAs are not expected to be conserved and this limits the ability of algorithms dependent on conservation to identify them. However, since DIANA-microT 3.0 algorithm accepts non conserved MREs it can successfully cope with viral miRNA sequences.

## Authors' contributions

MM and PA designed and developed the algorithm, performed the statistical analysis and drafted the paper. GLP contributed in the algorithm's implementation. MR participated in the algorithm's design and drafted the paper. TD, GG (Giannopoulos G.), TV and TS participated in the design and implementation of the web server database. GG (Goumas G.), EK, KK, NK, PT participated in the implementation of the algorithm's parallelization and contributed in the development of the online execution of the algorithm. VAS contributed in the web server design and development. PS helped to draft the paper and participated in the early development of the algorithm. AGH conceived of the study, and participated in its design and coordination and helped to draft the manuscript. All authors read and approved the final manuscript.
